# A bimodal extension of the Eriksen flanker task

**DOI:** 10.3758/s13414-020-02150-8

**Published:** 2020-11-11

**Authors:** Rolf Ulrich, Laura Prislan, Jeff Miller

**Affiliations:** 1 Department of Psychology, University of Tübingen, Schleichstr. 4, 72076 Tübingen, Germany; 2 Department of Psychology, University of Otago, Dunedin, New Zealand

**Keywords:** Conflict tasks, Eriksen flanker task, Auditory flankers, Bimodal stimulation

## Abstract

The Eriksen flanker task is a traditional conflict paradigm for studying the influence of task-irrelevant information on the processing of task-relevant information. In this task, participants are asked to respond to a visual target item (e.g., a letter) that is flanked by task-irrelevant items (e.g., also letters). Responses are typically faster and more accurate when the task-irrelevant information is response-congruent with the visual target than when it is incongruent. Several researchers have attributed the starting point of this flanker effect to poor selective filtering at a perceptual level (e.g., spotlight models), which subsequently produces response competition at post-perceptual stages. The present study examined whether a flanker-like effect could also be established within a bimodal analog of the flanker task with auditory irrelevant letters and visual target letters, which must be processed along different processing routes. The results of two experiments revealed that a flanker-like effect is also present with bimodal stimuli. In contrast to the unimodal flanker task, however, the effect only emerged when flankers and targets shared the same letter name, but not when they were different letters mapped onto the same response. We conclude that the auditory flankers can influence the time needed to recognize visual targets but do not directly activate their associated responses.

Humans are able to attend selectively to particular information while ignoring sources of irrelevant information that impinge on the sensory system at any instant of time. The issue of to what extent unattended information is processed has been an important topic in research on selective attention, and subtle experimental tasks have been developed to address it (see Pashler, 1998).

One of the most influential experimental tools for investigating the cognitive mechanisms of selective attention was introduced by Barbara A. Eriksen and Charles W. Eriksen in 1974. In this choice reaction-time (RT) task, participants are usually asked to make a speeded response to a visual target item that is flanked by task-irrelevant items. For instance, the target letters S and H may require left and right responses, respectively. In congruent trials, the flanker items match the identity of the target item (e.g., SSS S SSS), whereas in incongruent trials, the flankers match the identity of the alternative target item (e.g., HHH S HHH). Responses are typically faster and more accurate in congruent than incongruent trials, and similar effects of irrelevant information on the processing of task-relevant information have been found even when participants have little awareness of flanker effects (Miller, 1987). Flanker effects are robust, which may explain their great popularity in studying visual attention and conflict processing more generally. In fact, some researchers have even observed flanker interference in tactile and auditory versions of the flanker paradigm (Chan et al., 2005; Craig, 1995; Wesslein et al., 2014).

Several researchers have attributed the starting point of the visual flanker effect to selection processes operating within the visual domain (Pashler, 1998). Although these processes select the target item for further processing, they are not entirely effective in blocking out the flankers, which will subsequently produce response competition at later processing stages (Eriksen, 1995).^1^ For example, the prominent spotlight theory views visual attention as analogous to a spotlight (Posner et al., 1980) or a zoom lens (Eriksen & St. James, 1986) that is circular or oval-shaped (for a review, see Cave & Bichot, 1999). According to this theory, only stimuli falling within the spotlight are selected for further processing. An attentional gradient is often thought to vary across the spotlight, with strong facilitation at its center and a gradual drop-off towards its edge (Cave & Bichot, 1999). As a result, the cued central region where the target will appear in a flanker task receives more attention than the flankers in the periphery. This attentional gradient also predicts an increasing interference when the flankers appear closer to the center of the spotlight, a prediction that has been confirmed (e.g., Eriksen & St. James, 1986). Another view of the spotlight assumes that its size is not constant but gradually shrinks in on the target location. A quantitative formalization of this dynamic view, the shrinking spotlight model (White et al., 2011), incorporates the notion of a narrowing spotlight within a diffusion process framework and provides reasonable quantitative accounts of empirical flanker data. Others have assumed that visual processing proceeds in two discrete stages, an early stage of low selectivity followed by a second stage of high selectivity (Hübner et al., 2010; Hübner & Töbel, 2012; Jonides, 1983). A quantitative formalization of this two-step process, the dual-stage two-phase model (Hübner et al., 2010), also provides very reasonable fits to data from the traditional flanker task.

It seems natural to assume that the traditional flanker effect is related to the specific properties of visual attention in accordance with spotlight models and that a failure of visual selection leads to later response competition in post-perceptual processing stages. In their original study, Eriksen and Eriksen (1974) included a condition in which targets and flankers differed perceptually but were still mapped to the same response. Even this condition facilitated RT, which strengthens the view that a meaningful proportion of the flanker effect arises at the level where responses are selected.

Such a post-perceptual locus of the flanker interference is also consistent with other interference effects produced in additional conflict tasks such as the Stroop task and the Simon task, and effects in these tasks are known to emerge even when targets and distractors stimulate different modalities. In the traditional Stroop task, the target stimulus is non-verbal (e.g., a color patch) whereas the distractor is verbal (e.g., a word). However, even spoken color words as distractors produce Stroop interference when participants are asked to name the color of a color patch (Cowan & Barron, 1987; Elliott et al., 2014; Hirst et al., 2019), that is, in a bimodal Stroop task. Similarly, bimodal interference effects have been also observed for the Simon task (Simon & Craft, 1970). These bimodal results strongly suggest that the dominant location of interference in all these conflict tasks is beyond a perceptual level—a parsimonious hypothesis that is in accordance with the principle of Occam’s razor. For example, the diffusion model of conflict tasks (Ulrich et al., 2015) is consistent with the hypothesis that distractor information impairs the processing of relevant information outside the perceptual system, that is, at a post-perceptual decisional level.

So far, no study has addressed flanker effects with letters as targets and distractors in a crossmodal extension of the standard Eriksen task. Nevertheless, crossmodal congruency effects akin to flanker effects have been reported for non-linguistic stimuli. In a pioneering study, Frings and Spence (2010) asked participants to discriminate between temporal patterns presented to their ears, eyes, or hands. For example, in one experiment, a single temporal pattern lasted 600 ms and consisted of a series of 30 ms intervals, with a pulse on or off within each interval. In each trial, the target pattern was presented to one modality, while a distractor pattern could appear in the same modality or in another modality. In congruent trials, the target and distractor patterns had the same rhythms, while the rhythms were different in incongruent trials. The study revealed crossmodal congruency effects on both RT and error rates, with longer RTs and less errors in congruent than in incongruent trials. It is not clear to what extent these congruency effects reflect mechanisms similar to those involved in the standard flanker task, however, for several reasons. First, the rhythm task required discrimination among arbitrary, unpracticed, experimenter-defined stimulus sets differing only in low-level sensory features, whereas the Eriksen task involves discrimination between highly overlearned letter stimuli. Second, the RTs and error rates in their study were strikingly high; mean RTs were generally larger than 1500 ms and error rates often larger than 20%—values that are at least double those typical of the Eriksen task. Third, Frings and Spence (2010) did not relate their congruence effects to those observed with letter flankers, so they did not conduct RT distributional analyses, as is now common with the Eriksen task. Nonetheless, as will be considered further in the General Discussion, comparison of these crossmodal congruency effects with those observed in flanker tasks may be informative.

In another experimental setting to examine crossmodal congruence effects (for a review see Merz et al.,, 2020), participants are initially requested to respond to various audiovisual stimulus pairs with a particular response, that is, during this first experimental part, they are trained to map bimodal stimuli to certain response alternatives, such as a red light combined with a high tone to one response alternative while a blue light combined a with low tone to the other alternative. In the second part of the experiment, potential crossmodal interference effects between the bimodal stimuli from the first phase are assessed. Participants are now requested to discriminate between unimodal stimuli (e.g., red versus blue light) while the tones from the first phase serve as distractors. In these studies faster responses and fewer errors are observed when a distractor was previously associated with the target stimulus (e.g., high tone with red light) than when it was associated with the alternative target (e.g., low tone with red light; Jensen et al.,, 2019; Jensen et al.,, 2020; Merz et al.,, 2019). Note, however, that these congruence effects are based on previously learned S-R associations that are no longer relevant to the task in the second part of their experiment, whereas congruence effects in the Eriksen task involve the currently active S-R mapping.

The present study introduces an alternative variant of the crossmodal flanker task. The stimuli (i.e., targets and flankers) in this task are letters as in Eriksen’s classic flanker task, which allows a direct comparison of this crossmodal flanker version with Eriksen’s classic unimodal task. It seemed conceivable to us that participants could reject auditory letter flankers because higher cognitive processes must be involved in the processing of linguistic information compared to color and tone frequency as in the aforementioned crossmodal variants of the flanker task (e.g., Posner & Mitchell, 1967). We conducted two experiments to investigate possible interactions between visual target letters and auditory flankers. Experiment 1 assessed whether a bimodal flanker effect can be established. Experiment 2 examined more closely the locus of the flanker effect observed in Experiment 1. We not only analyzed mean RTs and response accuracy but also assessed how these measures depend on response speed by computing Vincentized RT distributions and conditional accuracy functions.

## Experiment 1

This experiment emulates a traditional flanker task with auditory instead of visual flankers. The task-relevant targets are the letters H and S and are presented visually, whereas the auditory flankers H and S are presented via loudspeakers. Since the auditory flankers may take some time until they develop a congruency effect, we presented the flankers either 0, 250, or 500 ms before the onset of the visual target.

### Method

#### Participants

In the traditional flanker task, usually 6–28 participants are employed (Servant & Logan, 2019). To ensure high statistical power, we chose to run 36 participants. Specifically, if one proceeds from a medium effect size, *d* = 0.5, this sample size implies a statistical power of 80% for a paired *t* test with *α* = 0.05 (two-sided test). All participants (31 female and five male, mean age 20.1 years) were recruited from the University of Tübingen; they received course credits and provided written consent.

#### Stimuli and apparatus

The experiment was conducted in a noise-shielded cabin. The target letters H and S (Font Arial, 1.7 cm high and 1.2 cm wide) were displayed in white on a gray background in the middle of a CRT monitor in front of the participant (viewing distance 55–60 cm). A white fixation cross (1.2 cm × 1.2 cm) was used to mark this position and to indicate the beginning of an experimental trial.

The auditory flankers were presented via loud speakers that were positioned beside the computer monitor. These flankers were the letters H and S, spoken in German by a male, and they were obtained from the platform *freesound* https://freesound.org/people/reinsamba/sounds/69247/. The duration (500 ms) of each letter’s soundtrack was equated by using the open source audio editor *Audacity.* The loudness of these flankers at the ear was approximately 66 dB SPL.

Responses were made with the left and right index fingers on a German QWERTZ keyboard using the left and right control keys.

#### Procedure

A single trial started with the presentation of the fixation cross for 300 ms. With the offset of this cross, the soundtrack with the spoken flanker letter was started. The visual target letter appeared after a stimulus onset asynchrony (SOA) of 0, 250, or 500 ms. The response of the participant terminated the presentation of the target and the flanker soundtrack. In case of a wrong response, the word “falsch” (wrong) appeared for 2 s in red on the screen. After a correct response or after the feedback in case of a wrong response, the next trial started after 1 s.

The main session consisted of 720 trials. These trials were separated in 12 blocks with 60 trials each. Every experimental condition (3 SOAs × 2 flankers × 2 targets) appeared five times during a single block. The order of all trials was randomized for each block. Before the main session, each participant received 12 practice trials that included each condition. Each new block was initiated by the participant when she or he felt ready to proceed. The duration of the whole experiment was approximately 45 min. Participants were instructed to respond to visual stimuli and to ignore the auditory information. Half of the participants responded to H and S with the left and right index fingers on the left and right shift keys, respectively. For the remaining participants, this S-R mapping was reversed.

### Results and discussion

The data of three participants were discarded from data analysis because their overall response error rates exceeded 10% (i.e., 12.36, 12.22, and 16.29%).^2^ RTs outside the range of 150–1500 ms (0.09% too fast, 0.39% too slow) were also excluded from all analyses. Trials with incorrect responses (2.76%) did not enter the analyses of RT results. Percentage of correct responses (PC) and RT were subjected to separate ANOVAs and when appropriate corrected for violations of sphericity by the Greenhouse and Geisser (1959) procedure.

**Fig. 1:**
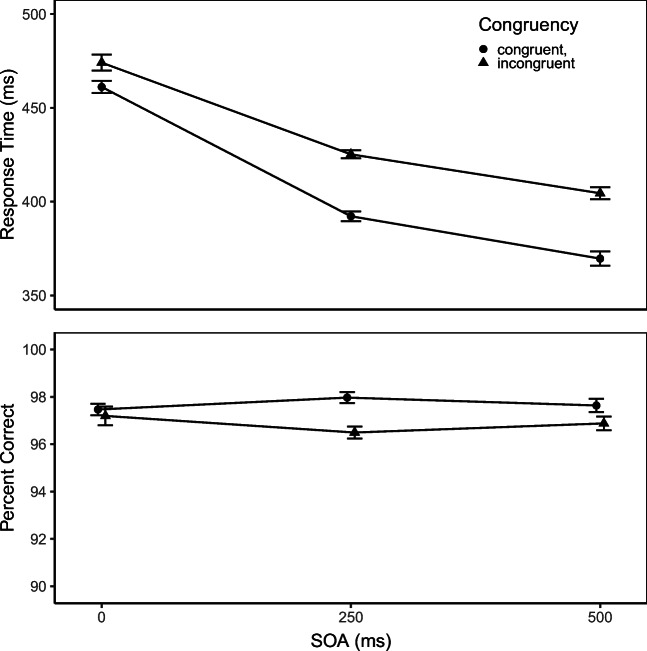
Mean reaction time (*upper panel*) and percentage of correct responses (*lower panel*) as a function of stimulus onset asynchrony (SOA) and congruency. The *error bars* reflect ± 1 SE, where SE denotes the within-subject standard error of mean computed according to Cousineau (2007) with the correction suggested by Morey (2008)

Figure 1 displays mean RT and PC as function of SOA and Congruency. PC was higher in the congruent condition (97.7%) than in the incongruent one (96.9%), *F*(1,32) = 9.63, *p* < .01, *η*^2^ = 0.23. Neither SOA, *F*(2,64) = 0.07, *p* = .94, *η*^2^ < 0.01 nor SOA×Congruency, *F*(2,64) = 2.57, *p* = .08, *η*^2^ = 0.07, produced a statistically significant effect on PC. RTs were longer on incongruent trials (435 ms) than on congruent ones (408 ms), *F*(1,32) = 125.45, *p* < .01, *η*^2^ = 0.80. Thus, the RT and PC results both demonstrate that auditory letter flankers affect the processing of the visual target letters. RTs decreased with increasing SOA, *F*(2,64) = 186.85, *p* < .01, *η*^2^ = 0.85. This main effect presumably demonstrates a response-unspecific effect of temporal preparation induced by the flankers (cf., Bertelson & Tisseyre, 1968; Müller-Gethmann et al.,, 2003), and this effect has also been reported for the standard flankers task (Hübner & Töbel, 2019). Moreover, there was a significant Congruency×SOA interaction, *F*(2,64) = 17.73, *p* < .01, *η*^2^ = 0.36, which reveals that the congruency effect on RT was larger when the auditory flankers preceded the target letter by more time, that is, the congruency effect was 13, 33, and 35 ms for SOAs of 0, 250, and 500 ms, respectively. A similar head start effect of the flankers has been observed in the unimodal flankers task (Hübner & Töbel, 2019; Mattler, 2003), although in those studies the flanker effect vanished at long SOA (i.e., 400 ms), suggesting that the time-course of this effect differs between auditory and visual flankers.

**Fig. 2:**
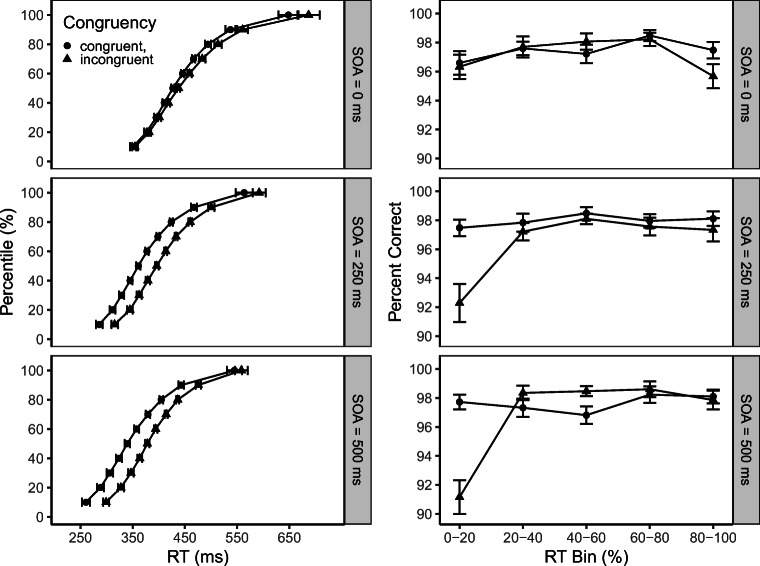
Vincentized cumulative response time distribution (*left column*) and bin percentage of correct responses versus bin mean RT (*right column*) as a function of stimulus onset asynchrony (SOA) and congruency. *Error bars* reflect ± 1 within-subject SE

Figure 2 traces the time-course of RT (left column) and PC (right column) under the different experimental conditions. First, for each participant and each combination of Congruency and SOA, RT values were determined that correspond to the *p*-th percentile (*p* = 5,15,…,95*%*). These percentile values of RT were then averaged across participants (i.e., Vincentizing) and the resulting cumulative RT distributions are displayed in the figure. The percentile values were also submitted to an ANOVA with factors percentile, SOA, and congruency. A significant interaction of percentile, SOA, and Congruency, *F*(18,576) = 5.10, *p* < .01, *η*^2^ = 0.14, indicates that the pattern of the congruency effect across the RT distribution was modulated by SOA. As can be seen in the figure, when the target appeared simultaneously with the onset of the auditory flanker, some time was needed to build up an interference effect; consequently, the congruency effect increased with percentile for SOA = 0. When the target was presented after the onset of the flanker, however, the interfering effect of the flanker was already present when the processing of the target started. A similar modulation of the congruency effect by SOA has been reported for the unimodal flanker effect (Hübner and Töbel, 2019; Mattler, 2003).

Second, RT distributions of each participant were sliced into five RT bins, that is, 0–20%, 20–40%, 40–60%, 60–80%, and 80–100%. For all trials within a bin, the percentage of correct responses was computed (i.e., conditional accuracy). The significant SOA×Bin×Congruency interaction on PC shows that response errors occurred mostly for fast responses, especially when the auditory flanker preceded the target by a longer SOA, *F*(8,256) = 4.15, *p* < .01, *η*^2^ = 0.12; this effect is also common for the unimodal flanker task (e.g., Hübner & Töbel, 2012; Ulrich et al.,, 2015).

In conclusion, concerning the time-course of the interference effect, this additional analysis reveals a pattern for the bimodal flanker task qualitatively similar to the one seen in the unimodal flanker task with varying SOA (Hübner & Töbel, 2019; Mattler, 2003). Specifically, the congruency effect appears to change from an increasing function at SOA = 0 to a horizontal or even sightly decreasing function at longer SOAs.

## Experiment 2

Experiment 1 indicates that auditory flankers produce effects similar to those of visual flankers in the standard unimodal Eriksen task. In Experiment 2 we included a further congruency condition analogous to a condition in the original study by Eriksen and Eriksen (1974). Specifically, in some experiments these authors included trials in which targets and flankers were perceptually different but were still mapped to the same response. They observed that RTs were still facilitated in this condition—though less facilitated than with identical flankers—and consequently concluded that a meaningful proportion of the flanker effect must arise in post-perceptual processing stages. Experiment 2 assessed whether this conclusion would generalize to the bimodal flanker task.

### Method

#### Participants

A fresh sample of 36 participants was recruited (24 females and 12 males, mean age 24.83 years). Two participants were replaced because they made more than 10% errors.

### Stimulus and apparatus

The apparatus and the stimuli were the same as in Experiment 1 except that we extended the stimulus set by including the additional letters Q and V. Thus, each letter of the set H, S, Q, and V was used as a visual target and as an auditory flanker.

### Procedure

The time-course of a single trial was nearly the same as in Experiment 1 with the following three exceptions. First, the visual target letter always appeared 500 ms after the onset of the spoken flanker. Second, for each participant, two letters were assigned to one response and the remaining two letters to the other response. Third, three different types of congruency conditions were realized in this experiment, that is, a flanker could be congruent with the target and the response (perceptual-congruent), only congruent with the response (response-congruent), or response incongruent. Specifically, target letters and flankers letters were factorially combined, resulting in 16 combinations and thus 16 different types of trials. In four combinations, the target letter and the flanker letter were identical. In four combinations, the flanker and target letter were different but the flanker information was nonetheless congruent with the response. In the remaining combinations, the target and the flanker were associated with incongruent responses.

As before, the main session consisted of 720 trials and these trials were again separated in 12 blocks with 60 trials each. Every experimental condition (4 flankers × 4 targets) appeared five times during a single block. The assignment of the letters to targets and flankers were counterbalanced across participants. For the data analysis, each trial was categorized as perceptual-congruent, response-congruent, or incongruent.

### Results and discussion

The overall percentage of correct responses was 96.8%. As in the previous experiment, trials with RTs outside the range 150–1500 ms (0.05% and 0.88%) and incorrect responses were excluded from all RT analyses. Separate one-way ANOVAs with conditions ‘perceptual-congruent’, ‘response-congruent’, and ‘incongruent’ were performed for PC and RT.

**Fig. 3:**
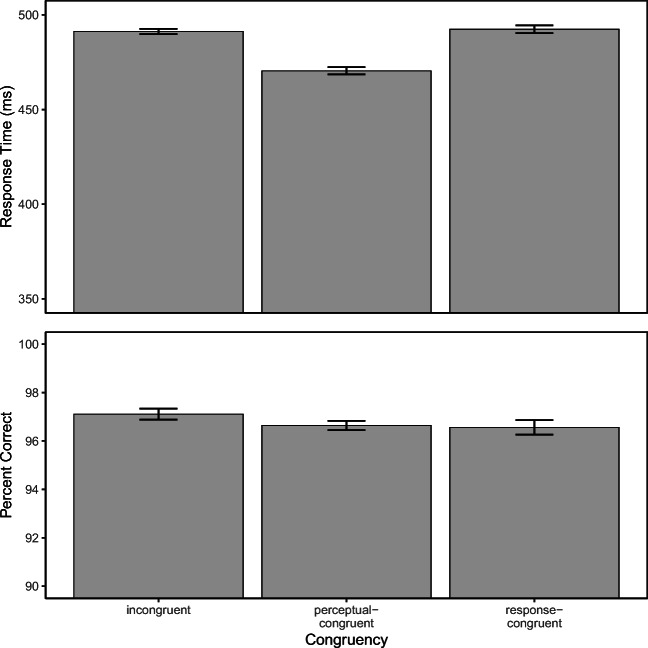
Mean reaction time (*upper panel*) and percentage of correct responses (*lower panel*) as a function of congruency. The *error bars* reflect ± 1 within-subject SE

Figure 3 depicts mean RT and PC for each congruency condition. ANOVA on PC revealed no statistically significant difference between the three conditions, *F*(2,70) = 1.45, *p* = .24, *η*^2^ = 0.04. However, the three conditions produced different mean RTs, *F*(2,70) = 47.05, *p* < .01, *η*^2^ = 0.57; although RT was virtually identical in response-congruent (492.4 ± 2.1 ms) and incongruent trials (491.3 ± 1.4 ms), there was a speed advantage in perceptual-congruent trials (470.5 ± 1.9 ms).^3^ This result is consistent with the hypothesis that auditory flankers facilitate the processing of visual targets when their letter names are identical. However, the results are at variance with the idea that auditory flankers speed-up late processes as there was virtually no processing benefit when auditory flankers and targets matched the same response. Accordingly, the conclusion that a significant proportion of this bimodal flanker effect resides in late processing stages is not supported by the present results.

**Fig. 4:**
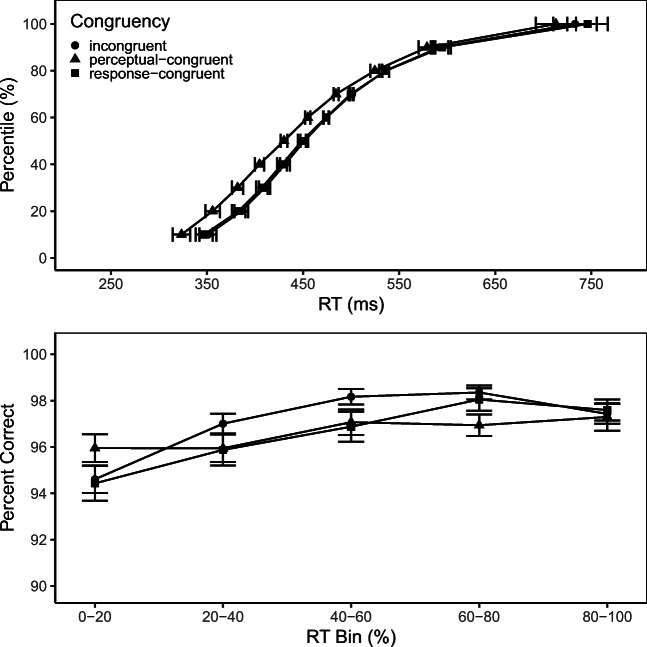
Vincentized cumulative response time distribution (*upper panel*) and bin percentage of correct responses versus bin mean RT (*lower panel*) as a function of congruency. *Error bars* reflect ± 1 within-subject SE

The upper panel in Fig. 4 shows the Vincentized cumulative RT distribution for each congruency condition. As one expects, RT increases with percentile, *F*(9,315) = 173.54, *p* < .01, *η*^2^ = 0.83, and it depends on condition, *F*(2,70) = 45.24, *p* < .01, *η*^2^ = 0.56. However, there was only a marginally significant interaction of percentile and congruency, *F*(18,630) = 2.26, *p* = .08, *η*^2^ = 0.06, in the direction that the effect of perceptual-congruency diminished for slower responses, which seems to resemble the RT result for SOA= 500 ms in Experiment 1. The lower panel illustrates how PC depends on response speed within each condition. As the figure suggests, slower responses tended to be more accurate, *F*(4,140) = 11.62, *p* < .01, *η*^2^ = 0.25. Consistent with the above analysis on PC, congruency did not produce a significant effect on PC, *F*(2,70) = 1.46, *p* = .24, *η*^2^ = 0.04, nor did the interaction of RT bin and congruency yield a reliable effect on PC, *F*(8,280) = 1.61, *p* = .15, *η*^2^ = 0.04. In summary, these additional analyses indicate that the effect of perceptual congruency is most consistent with a decreasing delta function and that response accuracy increases with RT, presumably reflecting a micro-speed–accuracy trade-off (Pachella, 1974), which, however, did not interact with congruency condition.

## General discussion

The present experiments demonstrate that flanker effects can also be induced in a bimodal flanker task with letters as visual targets and auditory flankers rather than physical stimuli like color and tone frequency (Merz et al., 2020). However, the results of the second experiment support the notion that auditory flankers only influence the time required to recognize the visual targets but do not activate their associated responses, unlike the response-level effects of flankers found in unimodal visual studies. Nonetheless, the present results resemble the ones observed in the unimodal task (Hübner & Töbel, 2019; Mattler, 2003) in that the effect of the flankers is rather short-lived because it tends to affect fast but not slow responses.

The present results are also consistent with bimodal spatial interference effects observed in the Simon task (Simon & Craft, 1970) and in cued modality-switching paradigms (Lukas et al., 2010; Tomko & Proctor, 2017), as well as with bimodal interference effects observed in Stroop paradigms where the irrelevant stimuli share features with the responses (Elliott et al., 2014; Hirst et al., 2019). However, whereas the visual flanker effect seems to be a combination of letter-specific and response-specific activation, the auditory flanker effect seems to be purely letter-specific. Thus, auditory flankers may also provide a way to study perceptual interactions uncontaminated by response activation effects.

The present results cannot easily be explained within typical “logogen”-type models (Morton, 1969). In such models, stimuli activate abstract (i.e., modality-independent) letter codes, whether these stimuli are delivered acoustically or presented visually. These codes then activate the responses with which they are associated. This apparently happens automatically, as is indicated by various conflict effects. Such models must predict response-congruent facilitation for auditory flankers, because these clearly do activate letter codes, as is indicated by the facilitation from the perceptual-congruent flankers.

Hence, the most surprising finding of the present study is that bimodal flankers produced perceptual-congruent effects but did not also produce response-congruent effects like their unimodal counterparts. The differing unimodal and bimodal effects might be conceptualized in terms of two processes: (a) a perceptual process during which the letter is recognized, and then (b) a response activation process which activates a response finger. In the bimodal task, apparently the auditory flanker only influences process (a), making it faster to recognize the visual letter in perceptual-congruent trials. This kind of perceptual-level interaction is also present with visually identical flankers in the unimodal task, but in that task there is also an additional contribution from process (b). The contribution from this later process in the unimodal task is responsible for the difference between the response-congruent and response-incongruent conditions in that task (Eriksen & Eriksen, 1974). However, it remains unclear to us why there is no analogous late contribution in the bimodal task, although our results clearly suggest no such late contribution. Future studies are required to resolve this unexpected finding.

The present results also help to inform models of conflict tasks. As was mentioned in the introduction, the standard flanker effect has often been related to a perceptual filtering mechanism and subsequent response competition. For example, one prominent explanation of the standard flanker effect involves the notion of a shrinking spotlight (Posner & Snyder, 1975). Indeed, a quantitative formalization of this spotlight notion provides a reasonable fit to empirical flanker data (White et al., 2011), as do quantitative models that posit an early locus of the flanker effect (Hübner et al., 2010). As mentioned in the preceding paragraph, the results of Experiment 2 are consistent with such a perceptual account of the bimodal flanker effect. Nevertheless, the results of the traditional flanker task also suggest that a meaningful proportion of the flanker effect arises in post-perceptual processing stages for unimodal flankers. Therefore, quantitative accounts assuming an exclusively perceptual locus of this effect are incomplete. Although congruency effects seem to arise at different processing stages, the mechanisms producing these effects may operate according to the same principle (Ulrich et al., 2015).

In summary, the present experiments have shown that the standard flanker effect of the traditional Eriksen flanker task can also be extended to a bimodal analog of this task with visual target letters and auditory irrelevant letters. Thus, bimodal conflict effects not only arise in the Stroop and Simon tasks but also within the prominent flanker task that was introduced by Barbara A. Eriksen and Charles W. Eriksen in 1974. Although all these effects are behaviorally similar, they seem to emerge at different stages within the processing stream from input to the associated response—the similarities and differences between unimodal and bimodal flanker effects clearly document the complexity in understanding these underlying conflict processes.

## Footnotes

1 According to Eriksen (1995, p. 111), response competition is not necessarily due to failures of selective attention. In fact, he favored the view that “attention is not only selection of the relevant stimulus but also the concurrent inhibition of the irrelevant competing stimulation” (p. 112) and thus mainly assumed that the primary cause of response competition is “reciprocal inhibition in the response system” (p. 111). To the best of our knowledge, all explanations of the flanker effect imply some sort of response competition, even contemporary quantitative models of the flanker effect.

2 Including these three participants, however, would not meaningfully alter the results.

3 The SEs are within-subject standard errors (Cousineau, 2007; Morey, 2008).
